# Superconductivity Bordering Rashba Type Topological Transition

**DOI:** 10.1038/srep39699

**Published:** 2017-01-04

**Authors:** M. L. Jin, F. Sun, L. Y. Xing, S. J. Zhang, S. M. Feng, P. P. Kong, W. M. Li, X. C. Wang, J. L. Zhu, Y. W. Long, H. Y. Bai, C. Z. Gu, R. C. Yu, W. G. Yang, G. Y. Shen, Y. S. Zhao, H. K. Mao, C. Q. Jin

**Affiliations:** 1 Beijing National Laboratory for Condensed Matter Physics and Institute of Physics, Chinese Academy of Sciences, Beijing 100190, China; 2 Collaborative Innovation Center of Quantum Matters, Beijing, China; 3 HiPSEC, Department of Physics and Astronomy, University of Nevada at Las Vegas, Las Vegas, NV 89154-4002, USA; 4 High Pressure Synergetic Consortium (HPSynC) & High Pressure Collaborative Access Team (HPCAT), Geophysical Laboratory, Carnegie Institution of Washington, Argonne, Illinois 60439, USA; 5 Center for High Pressure Science & Technology Advanced Research (HPSTAR), Shanghai, China; 6 School of Physical Sciences, University of Chinese Academy of Sciences, Beijing 100190, China

## Abstract

Strong spin orbital interaction (SOI) can induce unique quantum phenomena such as topological insulators, the Rashba effect, or p-wave superconductivity. Combining these three quantum phenomena into a single compound has important scientific implications. Here we report experimental observations of consecutive quantum phase transitions from a Rashba type topological trivial phase to topological insulator state then further proceeding to superconductivity in a SOI compound BiTeI tuned via pressures. The electrical resistivity measurement with V shape change signals the transition from a Rashba type topological trivial to a topological insulator phase at 2 GPa, which is caused by an energy gap close then reopen with band inverse. Superconducting transition appears at 8 GPa with a critical temperature *T*_*C*_ of 5.3 K. Structure refinements indicate that the consecutive phase transitions are correlated to the changes in the Bi–Te bond and bond angle as function of pressures. The Hall Effect measurements reveal an intimate relationship between superconductivity and the unusual change in carrier density that points to possible unconventional superconductivity.

Studies on the systems of strong spin orbital interactions (SOI), which play important roles in forming novel quantum states, intensively grown^1^,^2^,^3^,^4^,^5^,^6^,^7^,^8^. A classical SOI phenomenon is the Rashba effect, which lifts the electron spin degeneracy with broken inversion symmetry^9^,^10^. The Rashba effect is a momentum dependent splitting of spin bands in a no-centrosymmetric system. Other classical examples of SOI include topological quantum states such as topological insulators (TIs), wherein the bulk insulator state coexists with the robust metal edge (surface) state protected by topological invariance. The evolution of the Rashba system into a TI can provide an effective pathway to understand the mechanisms of these novel quantum states and their intrinsic connections, as well as provide opportunities for spintronic applications^11^. More importantly, SOI phenomena such as TIs or the Rashba effect can be utilized to realize a p-wave superconductor^3^,^12^. Similar to TIs, topological superconductors are expected to have gapless edge (or surface) states that can host long sought elusive Majorana fermions. Such unconventional superconductors are potentially important for new quantum computers^7^,^8^.

Rashba polar semiconductor BiTeI hosts the largest known spin split band from its bulk polar atomic configuration^9^ with a Rashba parameter of 3.85 eVÅ at ambient conditions. It was recently theoretically predicated that BiTeI can be transformed from a topological normal state into a topological no trivial insulator by band inverse at high pressures^10^. The prediction received immediate experiments follow-up through observation of band close up then reopen & inverse as detected by IR spectroscopy^13^. High pressure can be a powerful method to generate new quantum states since it perishes the by products such as disorders or impurities introduced by chemical doping. Transport measurements at high pressures are especially effective in unveiling quantum state evolution in the SOI system since the TI compounds of Bi_2_Te_3_^14^, Sb_2_Te_3_^15^, or Bi_2_Se_3_^16^ become superconductive at high pressures.

This paper reports on the discovery of superconductivity in BiTeI induced by pressure that borders the transition from a Rashba type topological trivial state to a topological insulator state. Structure evolutions, including local structure distortion at high pressures, are investigated by Raman spectroscopy in conjunction with high pressure synchrotron X-ray diffraction.

## Results and Discussion

### High-pressure electrical transport properties of BiTeI single crystal

**Figure 1:**
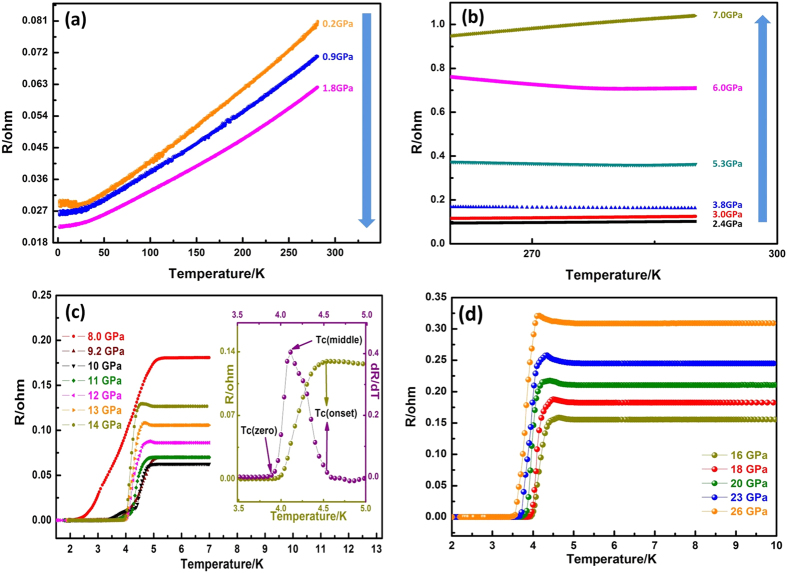
(**a,b**) Resistance of the BiTeI single crystal as function of temperature in the low pressure phase shows the topological quantum phase transition at ~2 GPa; (**c,d**) Resistance of the BiTeI single crystal as function of temperature in the high pressure range of 7 GPa to 26 GPa, which shows superconductivity at 5.3 K at 8.0 GPa. The inset of (**c**) is an enlargement of resistance versus temperature around the superconducting region at 14 GPa; the differential of resistance over temperature (dR/dT) shows the onset, middle, and zero transition temperatures, respectively.

The ambient phase of BiTeI crystallizes into a non-centrosymmetric layered structure of the trigonal space group *P*3*m*1 with lattice parameters of *a* = 4.3392 Å and *c* = 6.854 Å^10^,^17^. Figure 1 shows the resistance evolution of the BiTeI single crystal as functions of pressure and temperature. The low resistance of the BiTeI crystal is generally caused by additional conductivity by certain defects mentioned in ref. 9. The BiTeI crystal resistance decreases with pressure in the region lower than 2 GPa as shown in Fig. 1(a). However, the resistance increases to a maximum with pressure at ~7 GPa upon crossing the critical pressure^10^,^13^ of ~2 GPa as shown in Fig. 1(b). When the pressure reaches above 8 GPa, superconductivity appears with an onset superconducting transition temperature *T*_*C*_^(onset)^ of 5.3 K, where the value of *T*_*C*_ is defined using the method^14^ based on the differential of resistance over temperature as shown in the inset of Fig. 1(c). The *T*_*C*_^(onset)^ value decreased to 4.5 K at 14 GPa with a rate *dT*_*C*_*/dP* = −0.14 K/GPa and then reached approximately 4.1 K with a non-linear decrease as shown in Fig. 1(c) and 1(d), respectively.

**Figure 2:**
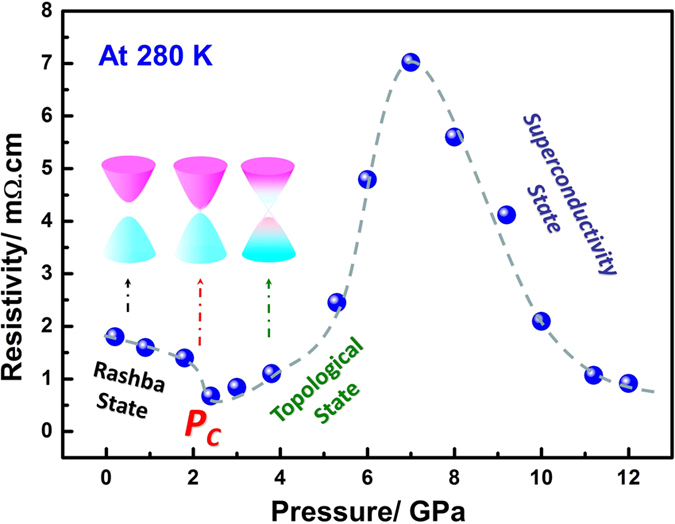
Resistivity of the BiTeI single crystal at 280 K and in the pressure range of 0 GPa to 12 GPa. The transition from the Rashba type topological trivial state to the topological non trivial quantum state at *P*_*C*_ ~2 GPa is indicated by the minimum resistivity level. Insets show schematics of the energy band inversion across the topological quantum phase transition. Magenta denotes the conduction band, whereas cyan denotes the valence band. At *P*_*C*_, the surface metallic state was represented by the Dirac Cone, which comes from the inversion of the Bi 6_*pz*_ orbital and Te/I-5*pz* orbitals.

Figure 2 shows the resistivity of the BiTeI single crystal at 280 K as function of pressures. The resistance decreases with pressure as a result of the energy band gap changes that narrows to a critical pressure (*P*_*C*_) of ~2 GPa where the gap closes in the Rashba phase. Upon crossing the *P*_*C*_, the inversion and opening up of the Bi 6_*pz*_ orbital and Te/I-5*pz* orbitals leads to the semiconducting behavior as shown in Fig. 1(b). The emergence of the semiconducting state, which comes from the conduction and valence band reconstruction, is the feature of the band gap reopening^10^. This phenomenon results in a V shape change in resistivity across *P*_*C*_ as shown in Fig. 2.

**Figure 3:**
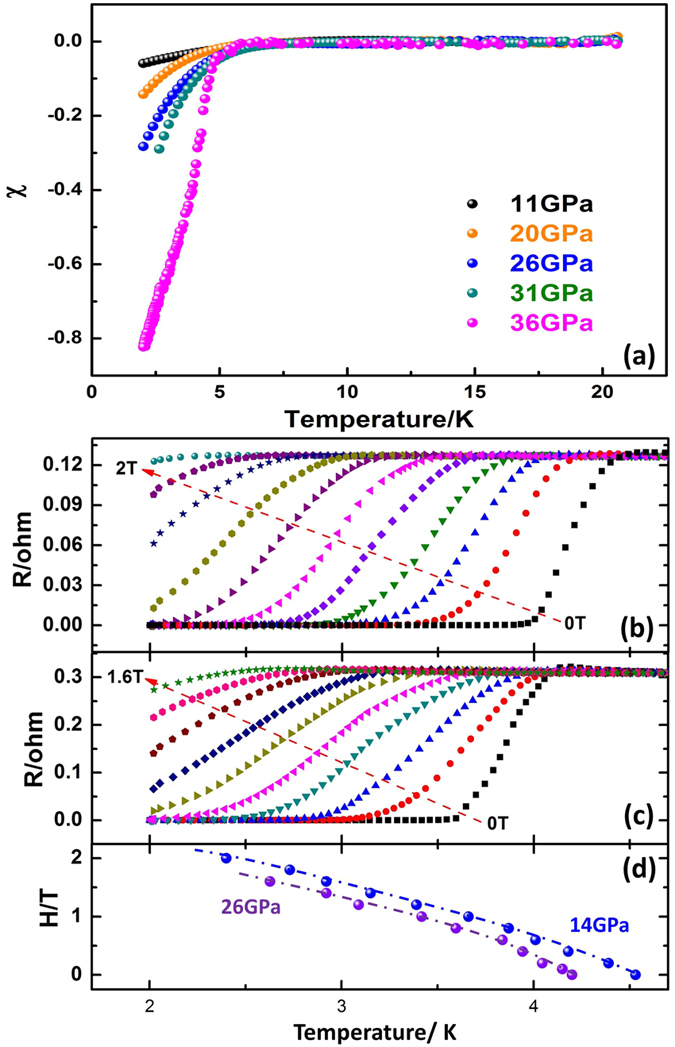
(**a**) a*c* magnetic susceptibility of BiTeI as a function of temperature at high pressure; (**b,c**) Magnetic field dependence of the superconductivity transition of the BiTeI single crystal at 14 GPa and 26 GPa with an applied magnetic field *H* perpendicular to the *ab* plane, and (**d**) the dependence of *T*_*C*_ on the magnetic field *H*.

**Figure 4:**
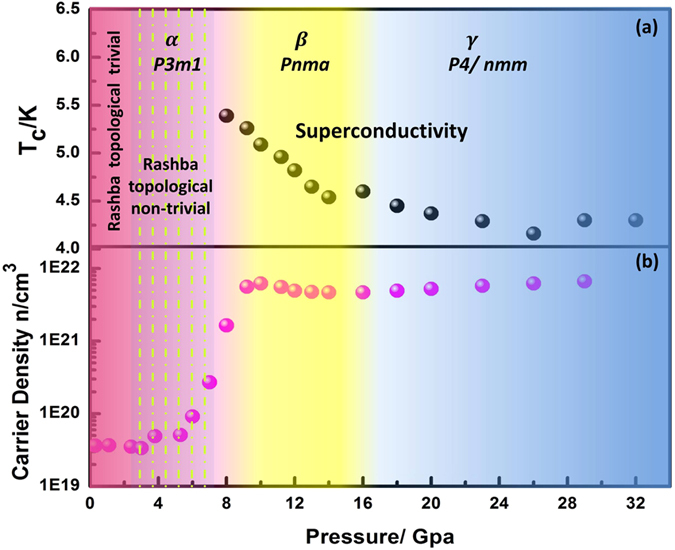
(**a**) Superconducting transition temperature (*T*_*c*_) and (**b**) n type carrier density of the BiTeI single crystal fixed at 30 K as function of pressures. The first superconducting transition appears at ~8 GPa, followed by extension into the region above ~16 GPa.

The superconductivity nature in the BiTeI single crystal was validated by the *ac* magnetic susceptibility measurement that results in transition with *T*_*C*_ at ~5 K that is similar to the conductivity measurements as shown in Fig. 3(a). Figure 3(b,c) shows superconducting transition as a function of the external magnetic field *H* perpendicular to the *ab* plane of the BiTeI single crystal. *T*_*C*_ decreases with the applied magnetic field *H*. The change in *T*_*C*_ with *H* is shown in Fig. 3(d). Using the Werthdamer–Helfand–Hohenberg formula^18^ of *H*_*C2*_*(0)* = *−0.691T*_*C*_ × *[dH*_*C2*_*(T)/ dT]|*_*T*_ = _*Tc*_, the upper critical field *H*_*c2*_ was estimated to be 2.9 T and 2.8 T at 14 GPa and 26 GPa, respectively, for the magnetic *H* perpendicular to *ab* plane, which are comparable to those of Bi_2_Te_3_^14^ or Sb_2_Te_3_^15^. The Hall Effect measurement on BiTeI was conducted at high pressure with a magnetic field *H* perpendicular to the *ab* plane of the sample up to 5 T at a fixed temperature (30 K) for each given pressure. The samples show n type carrier features in all pressure temperature ranges as shown in Fig. 4.

### Structure evolution of the BiTeI single crystal at high pressures

High pressure synchrotron X-ray diffraction of BiTeI was conducted (Fig. S1a) to trace the origination of the different quantum state transitions considering crystal structural evolutions. Rietveld refinement was performed for the X-ray diffraction patterns, and the refined volumes of all the phases are shown in Fig. S1(b). The first pressure induced structure transition was observed above 10.6 GPa, whereas the second structure transition was observed above 20 GPa as shown in Fig. S1(a).

Raman spectroscopy was conducted to obtain insights into the local structure evolution at high pressure levels (Fig. S3). The symmetry analysis indicates that BiTeI has four zone center vibrational modes at ambient conditions with the irreducible vibrational representation Γ = 2A_1_ + 2E^19^. Figure S3(c) shows that: (i) the intensity of the A_1_(1) vibrational mode is gradually suppressed and disappears at ~4 GPa at ambient phase, which has relevance with the electronic topological transition, although no sufficient theoretical evidence proves this condition; (ii) A_1_(2) and E(2) vibrational modes blue shift up to ~7.5 GPa, and a new mode M1 at ~136 cm^−1^ appeared above ~8.9 GPa, which should be the signatures for the first structure transition; (iii) all the three modes become softened above ~15.8 GPa, whereas a new mode M2 appeared at ~163 cm^−1^ that can be associated with the further high pressure induced structure transition.

The high pressure synchrotron X-ray diffraction and high pressure Raman spectroscopy experiments jointly indicate that the first pressure induced structure transition should be at 7.5 GPa to 8.9 GPa from the hexagonal *P3m1* (denoted as α BiTeI) ambient phase to the orthorhombic *Pnma* (denoted as β BiTeI) high pressure phase. The second structure transition should be at 15.8 GPa to 17.2 GPa from the orthorhombic *Pnma* high pressure phase to the tetragonal *P*_4_*/nmm* (denoted as γ BiTeI) high pressure phase^13^,^20^. The coordination of Bi cations is believed to take important role in tracing (tuning) the changes; their schematic views are shown in Fig. S2.

Figure 4 summarizes the global phase diagram of the quantum phase evolution of BiTeI, especially the relationship between the superconductivity temperature *T*_*C*_ **(a)** and carrier densities **(b)** as function of pressures. BiTeI is of n type carriers with a carrier density of 3.62 × 10^19^ cm^−3^ at ambient pressure as measured from the Hall coefficient similar to that in^9^. The carrier density changes slightly below 6.0 GPa. The carrier density above 6 GPa increases rapidly over three orders of magnitude that reaches approximately 6.23 * 10^21^ cm^−3^ at 10 GPa as indicated in Fig. 4(b). Consequently, the superconducting state is accompanied with a sharp increase in the carrier density. The increases in carrier density across phases α to β after the topological trivial to non-trivial transition in the Rashba phase is therefore expected to signal the pressure induced superconductivity. *T*_*C*_^(onset)^ decreases with the further pressure increase to 32 GPa.

The previous structure^17^ study indicates that a semi-ionic model with polar axis along the atomic stacking direction can be applied to describe the BiTeI structure at ambient phase that consists of a corrugated (BiTe)^+^ layer and I^−^ anions; however, Bi-I contacts are considered to be ionic^9^.

**Figure 5:**
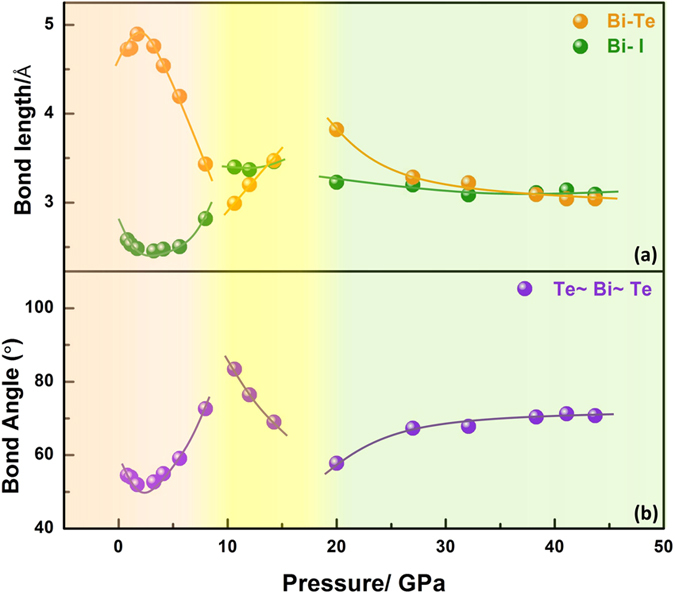
(**a,b**) Structure parameters about the bond lengths of Bi-Te and Bi-I, as well as the bond angle of Te~Bi~Te.

To understand the structural related origin of transport properties, we plotted the bond length of Bi-Te and Bi-I, the bond angle of Te~Bi~Te obtained from the Rietvield Refinements based on our experiments in Fig. 5.

Figure 5 shows that in phase α (ambient pressure phase), the bond length of Bi-I decreases with pressure to ~2 GPa, then increases sharply with pressure to ~8 GPa. The same results are observed for the bond angle of Te~Bi~Te. This scenario causes another V shape change across *P*_*C*_. The decrease in the Bi-I bond length with pressure to 2 GPa indicates an enhanced band width, which results into a gradual narrowing and closing of the band gap. The increase in these structural parameters with pressure to 8 GPa in turn leads to the gradual reopening of the band gap above 2 GPa. Signatures of V shape change of resistivity from transport measurements shown in Fig. 2 therefore well correlate to the evolution of respective structural parameters.

The BiTeI structure in phase β (high pressure phase 1) consists of positively charged (BiTe)^+^ ladders, where Bi-Te has weak Bi−Te covalent bonds surrounded by I^−^ ions^18^. The increase in the Bi-Te bond length with pressure can result in more ionic bonds of Bi-Te, which results in less localized behavior of electrons. The decreased bond angle of Te~Bi~Te with pressure compresses the (BiTe)^+^ ladders along the b-axis and suppresses the band gap. The above structure distortion can contribute to less semiconductive behavior and properly coincides with the resistivity decrease in this phase as shown in Fig. 2. Figure 4 shows that *T*_*C*_ decreases dramatically, whereas the carrier density maintains almost no change in the pressure range from 11 GPa to 20 GPa. This condition is inconsistent with conventional superconductivity, where *T*_*C*_ generally changes as function of carrier density^21^. However, the phonon frequency and electronic density of states *N(E*_*F*_) can be balanced to control *T*_*C*_as stated by BCS theory or the McMillan electron-phonon coupling formalism^22^. Therefore, anomaly phonon spectrum changes in this pressure range can be expected. The refined bulk modulus *B*_*0*_ and lattice volume *V*_*0*_ in phases *α* and *β* are comparable as shown in Fig. S1, which indicates that the structure of phase *β* can preserve a similar lattice property with phase *α*. Considering that superconductivity emerged at the border across the topological phases *α* to *β*, this condition implies that the superconductivity in this pressure range should result from a topological precursor^3^.

The bond length of the Bi-Te atoms in phase γ (high pressure phase 2) decreases, whereas the Bi-I bond length remains almost constant. The electronic band structure shows that BiTeI in this phase is metallic^18^. The *T*_*C*_ in this phase initially decreases when the bond length of the Bi-Te atoms decreases. *T*_*C*_ then increases slightly when the bond lengths of Bi-Te and Bi-I, as well as the Te~Bi~Te bond angle, remain almost non change. These observations indicate that the *T*_*C*_ variation correlates with the structure evolution at pressures. Figure S1b shows that the refined bulk modulus *B*_*0*_ in phase *γ* is much larger than that in phase *β*, whereas the refined *V*_*0*_ is much smaller than that of phase *β*, which indicates that the phase *γ* structure differs from that of phase *β*. The *ac* magnetic susceptibility measurement shows that the superconducting volume fraction increases with applied pressure, which signifies that the superconductivity transforms from the surface states at the border range in phases *α* to *β* to bulk nature in phase *γ*.

In summary, we successfully observed pressure induced consecutive transitions for a SOI compound from a Rashba-type topological trivial phase to a topological insulator followed by a classical quantum condensation (i.e., superconductivity). The evolutions properly correlate with the bonding parameter changes of the structures. The observed superconductivity from the topological precursor phase points to a possible unconventional nature.

## Methods

The BiTeI single crystals are grown utilizing the Bridgeman method. The Te powder, Bi, and I reagents are mixed together at a proper stoichiometric ratio. The mixtures are then grounded thoroughly in an agate mortar to ensure homogeneity. The mixtures are sealed in an evacuated quartz tube under 10^−4^ Pa and heated to 660 °C for 24 h, followed by slow cooling at a rate of 12 °C/h to 100 °C before quenching to room temperature.

The electronic transport properties of BiTeI at a low pressure range (<2 GPa) are measured through four probe electrical conductivity methods in a Piston Cylinder cell made of CuBe alloy. A piece of the BiTeI single crystal with a dimension of 2 mm * 1 mm * 0.15 mm was loaded into a Teflon-made vessel with Daphne oil loaded as the pressure transmitting medium. A manganese copper wire was placed near the sample as a pressure marker. The cell was placed inside a Maglab system with an automatic temperature control model. A semiconductor thermometer was mounted on the surface of the cell to monitor the temperature. The electronic transport properties of BiTeI with pressure higher than 2 GPa are measured through four probe electrical conductivity methods in a diamond anvil cell (DAC) made of CuBe alloy. The diamond culet is 300 μm in diameter. The Au wires with a diameter of 18 μm are used as electrodes. A T301 stainless steel gasket was compressed from a thickness of 250 μm to 40 μm, and then a hole at 150 μm in diameter was drilled. The cubic BN applied as an insulating layer was pressed into this hole. A small center hole at 100 μm in diameter was further drilled to serve as the sample chamber, where NaCl fine powder serves as a pressure transmitting medium and a piece of BiTeI single crystal with dimensions of 90 μm * 90 μm * 20 μm was loaded. A piece of ruby was loaded simultaneously as a pressure marker. The DAC was placed inside a Maglab system with automatic temperature control. A thermometer was mounted near the diamond to monitor the temperature. The high pressure Hall coefficients are measured utilizing the Van der Paul method.

The high pressure Raman experiments are conducted on single crystal BiTeI with dimensions of 60 μm * 60 μm * 20 μm with Renishaw Raman Spectroscopy with a laser wavelength of 532 nm and spectral resolution ~1 cm^−1^ in a DAC. Silicon oil was applied as the pressure medium. The diamond culet is 300 μm in diameter. The T301 stainless steel gasket was compressed from 250 μm to 40 μm, and a center hole at 140 μm in diameter was drilled. A small piece of ruby was placed aside the specimen to measure pressure.

High pressure synchrotron X-ray diffraction experiments are performed at 16 BMD, HPCAT of the Argonne National Laboratory with a wavelength of 0.4246 Å in a symmetric DAC with a culet at 300 μm in diameter. The T301 steel gasket was pre-indented from 300 μm to ~40 μm, and a center hole at 150 μm in diameter was drilled. The hole was compressed to 120 μm with neon loaded as a nearly hydrostatic pressure transmitting medium. Ruby balls are placed near the sample as pressure markers. The X-ray diffraction patterns are collected with a MAR 3450 image plate detector. The obtained 2D image plate patterns are converted to 1D 2*θ* versus intensity data utilizing the Fit2D software package^23^. Refinements of the measured X-ray diffraction patterns are performed by applying the GSAS + EXPGUI software packages^24^,^25^.

## Additional Information

**How to cite this article**: Jin, M.L. *et al*. Superconductivity Bordering Rashba Type Topological Transition. *Sci. Rep.* **7**, 39699; doi: 10.1038/srep39699 (2017).

**Publisher's note:** Springer Nature remains neutral with regard to jurisdictional claims in published maps and institutional affiliations.

## Supplementary Material

Supplementary Information
