# Supplementary material for: Superconductivity Bordering Rashba Type Topological Transition
Source: Sci Rep. 2017 Jan 4;7:39699. doi: 10.1038/srep39699 (PMC5209719; doi:10.1038/srep39699)
Supplement: Supplementary Information [file srep39699-s1.pdf]

Supplemental materials

Superconductivity Bordering Rashba Type Topological Transition

M.L. Jin[1], F. Sun[1,2], L.Y. Xing[1], S. J. Zhang[1], S. M. Feng[1], P. P. Kong[1], W.M.Li[1], X. C. Wang[1], J. L. Zhu[4], Y. W. Long[1], H.Y.Bai[1], C. Z. Gu[1], R. C. Yu[1], W. G. Yang[2,5], G.Y. Shen[5], Y. S. Zhao[1,4], H.K.Mao[2,5], C. Q. Jin[1,3]

1. Beijing National Laboratory for Condensed Matter Physics and Institute of Physics, Chinese Academy of Sciences, Beijing 100190, China

2. Collaborative Innovation Center of Quantum Matters, Beijing, China

3. Center for High Pressure Science & Technology Advanced Research (HPSTAR), Shanghai, China

4. HiPSEC, Department of Physics and Astronomy, *University of Nevada at Las Vegas, Las Vegas, NV 89154-4002, USA*

5. High Pressure Synergetic Consortium (HPSynC) & High Pressure Collaborative Access Team (HPCAT), Geophysical Laboratory, Carnegie Institution of Washington, Argonne, Illinois 60439, USA

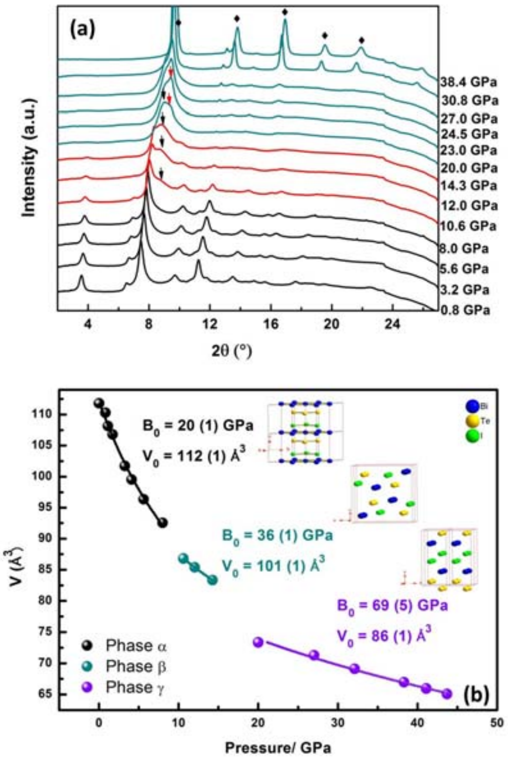

Figure S1 (a) X-ray diffraction patterns of BiTeI up to 38 GPa. The peaks marked with black arrows indicate phase transition from *P*3*m*1 to *Pnma*; red arrows correspond to *P4/nmm* phase. Solid diamonds are the diffraction peaks for *P4/nmm* phase. **(b)** the refined volume compression versus pressure of the BiTeI, where V_0 corresponds to the lattice volume of the ambient pressure, B_0 corresponds to the bulk modulus. The inset is crystal structures of BiTeI at three phases.

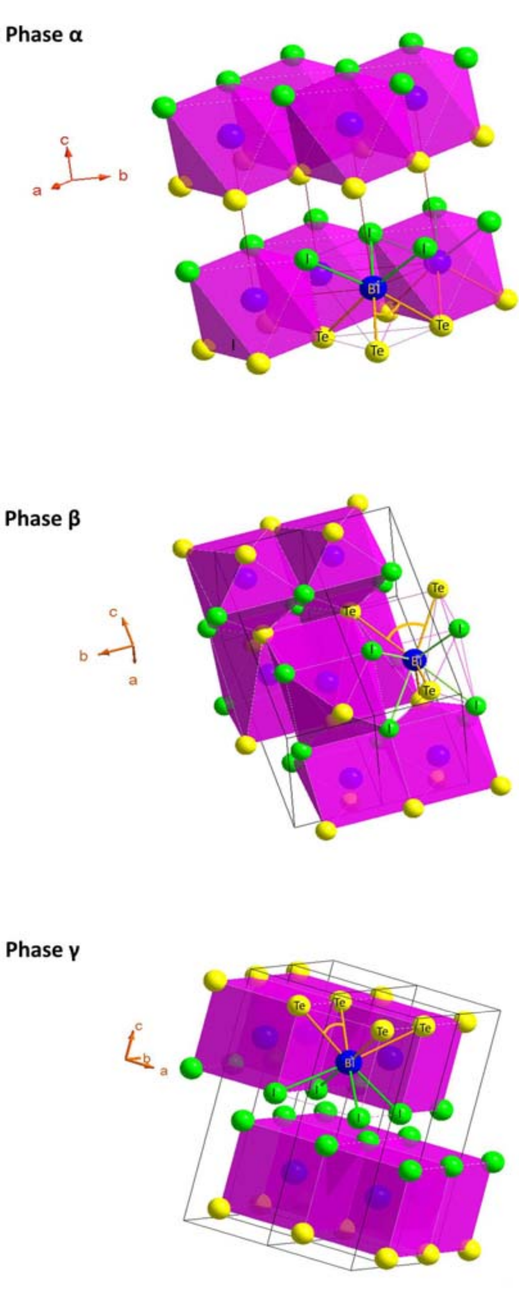

Figure S2 Schematic views of crystal structure for α, β and γ phase, respectively. In phase α, the Bi cation is coordinated by 3 Te and 3 I anions. In phase β and γ, the Bi cation is coordinated by 4 Te and 4 I anions. Bi, Te, I ions are denoted as blue, yellow and green spheres, respectively. The bond lengths of Bi-Te and Bi-I are denoted by yellow and green bars, respectively. The bond angle of Te～Bi～Te is denoted by yellow arc.

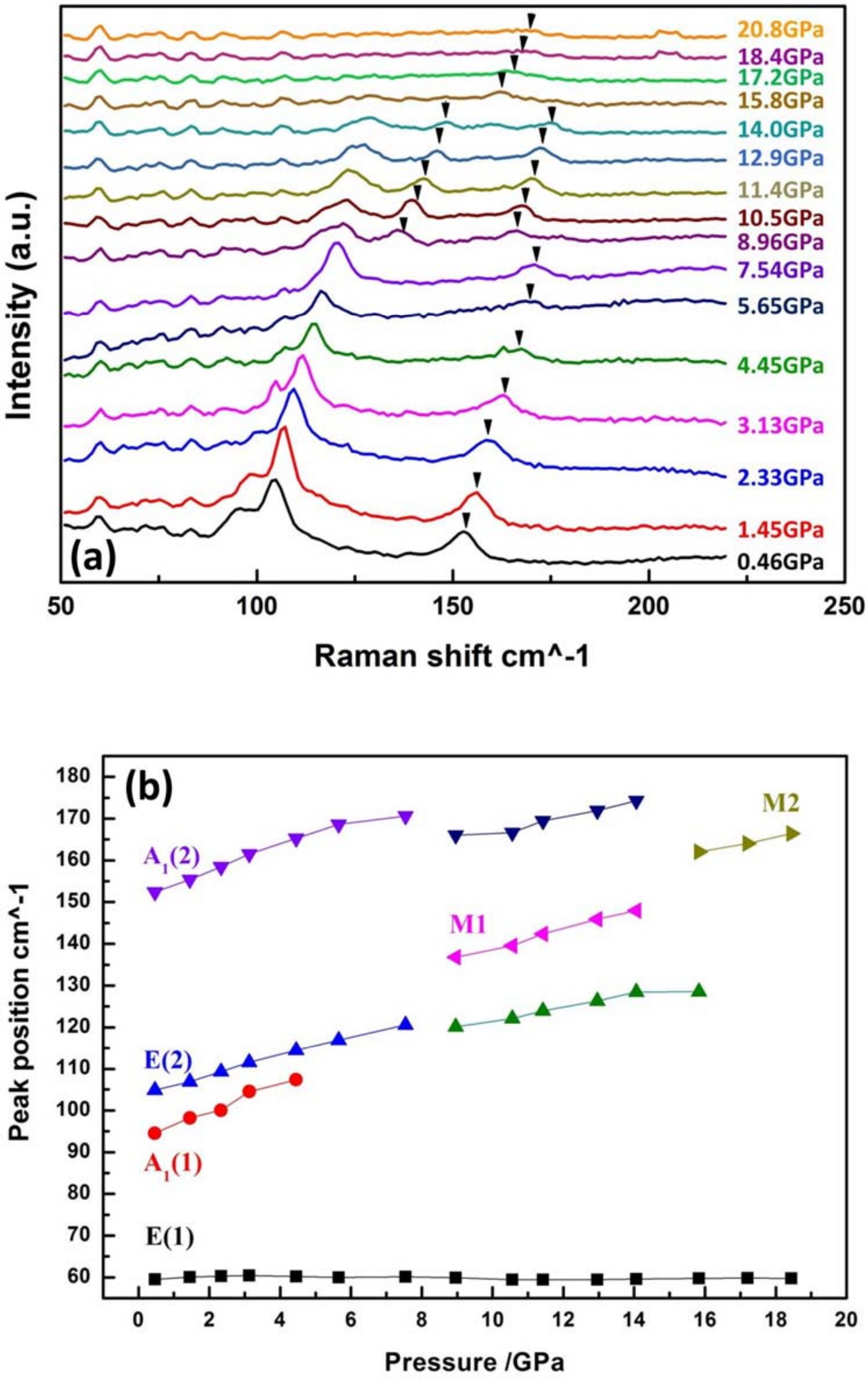

Figure S3 (a) Raman spectra of BiTeI as function of pressure. **(b)** Raman vibration modes evolutions as a function of pressure.
